# Unexpected death within 72 hours of emergency department visit: were those deaths preventable?

**DOI:** 10.1186/s13054-015-0877-x

**Published:** 2015-04-08

**Authors:** Hélène Goulet, Victor Guerand, Benjamin Bloom, Patricia Martel, Philippe Aegerter, Enrique Casalino, Bruno Riou, Yonathan Freund

**Affiliations:** Emergency Department, Hôpital Pitié-Salpêtrière, Assistance Publique- Hôpitaux de Paris (APHP), 47-83 Boulevard de l’Hôpital, 75013 Paris, France; Public Health Department, Hôpital Ambroise Paré, APHP, 9 avenue Charles de Gaulle, 92100 Boulogne-Billancourt, France; Emergency Department, Royal London Hospital, Whitechapel Road, London, E1 1BB UK; Université Versailles Saint-Quentin, Saint-Quentin en Yvelines, 55 avenue de Paris, Versailles, 78000 France; Emergency Department, Hôpital Bichat, APHP, EA 7334 REMES, Université Paris Diderot, Sorbonne Paris Cité, 46 rue Henri Huchard, 75877 Paris, France; Sorbonne Université, UPMC univ-Paris 6, UMRS INSERM 1166, IHU ICAN, 91 Boulevard de l’Hôpital, 75013 Paris, France

## Abstract

**Introduction:**

We aimed to determine the rate of preventable death in patients who died early and unexpectedly following hospital admission from the emergency department (ED).

**Methods:**

We conducted a retrospective multicenter study in four centers from the Paris metropolitan area. Inclusion criteria were medical patients who died in hospital within 72 hours of ED attendance and were not admitted to the intensive care unit (unexpected death). Exclusion criteria were limitations of care determined by treating physicians. The existence of a limitation of care decision was adjudicated by two independent chart abstractors. Preventable death was defined as death occurring as a result of medical error. For each selected patient with unexpected death, charts were examined for medical errors and rated on a 1 to 5 preventability scale (from very unlikely to very likely) for the preventability of the death. The primary endpoint was the likely preventable death, rated as 4 or 5 on the preventability scale.

**Results:**

We retrieved 555 charts; 47 unexpected deaths were analysed; 24 (51%) were considered as preventable. There was a median number of medical errors of two. The most common process breakdowns were incorrect choice of treatment (47% of patients) and failure to order appropriate diagnostic tests (38% of patients). The most common medical error was a severe delay or absence of recommended treatment for severe sepsis, which occurred in 10 (42%) patients.

**Conclusions:**

In our sample, more than half of unexpected deaths are related to a medical error, and could have been prevented.

## Introduction

The incidence and severity of medical errors in the emergency department (ED) is not accurately known [1]. This is in contrast to settings such as medical wards or the operating room [2-5]. Recent prospective studies suggest a rate of adverse events (AE) related to ED care of around 5 to 10% [6-8], of which half are preventable. Systematic detection of AEs or medical errors is complex and time consuming, and consequently the rate of AEs may be often underestimated [1]. The severity of AEs may be significant, 15 to 30% of them being life threatening [7,8]. Previous studies suggested that the incidence of early death related to ED care would be of 5 to 30 per 100,000 visits [9-11].

Reducing the incidence of all medical errors in the ED is essential, however, priority should be given to reducing the types of error that have the potential to cause AE, serious harm or death. To describe and understand mechanisms and factors that can lead to a preventable severe AE, several studies retrospectively analyzed emergency medical charts of discharged patients that sustained unexpected death [9,11-13]; or harm that precipitated malpractice claims [14].

The primary objective was to determine the rate of preventable death in patients that died unexpectedly within 72 hours of hospital admission from the ED. A secondary objective was to determine causes of preventable death including systemic error or process breakdown.

## Materials and methods

### Study design and settings

This was a retrospective study conducted between January 2007 and December 2011, in four urban academic EDs from Paris metropolitan area, France (Pitié-Salpêtrière, Bichat, Ambroise Paré, and Saint Antoine). The participating EDs have an annual census that ranges from 35,000 and 80,000 adult visits each year. The four participating centers are affiliated with Assistance Publique - Hôpitaux de Paris, and use the same administrative system, in which the time and date of admission and final disposition are automatically recorded (for example ‘discharged home on 11/02/2012 at 13:30’ or ‘in hospital death on 10/02/2012 at 13:30’). The study was approved by our institutional review board ‘Comité de Protection des Personnes - Paris Ile de France 6’ (Paris, France) without the need of informed consent.

### Primary endpoint

The primary endpoint of the study was preventable death. Preventable death was defined as death occurring as a result of medical error. We used the definition of the Institute of Medicine for the definition of an error: the failure of a planned action to be completed as intended, or the use of a wrong plan to achieve an aim [2].

### Secondary endpoint

The secondary endpoint was process error classification. Process error classification was reported according to the processes described below, and is adapted from previous literature [14].

We defined that different possible breakdown would occur in any of the following processes:

- ED arrival
- Nurse triage
- Medical history/physical examination
- Appropriate diagnostic tests
- Timing of test ordering
- Interpretation of test results
- Appropriate treatment
- Timing of treatment ordered/given
- Wait/monitoring
- ED disposition
- Handover
- Medical ward
- Operation room

### Selection of participants

The inclusion criteria were patients who were admitted into hospital from the ED, but not to high dependency or intensive care units (HDUs, ICUs), and died within 72 hours. Patients who died in the ED were considered to have been admitted, and were included in the analysis.

The process of identification of cases took several steps. Initially, a database was interrogated to identify admited patients with early death and no ICU/HDU admission. Of those, cases were randomly selected to undergo a first chart abstraction that was designed to select patients who may have died unexpectedly. Finally, a second chart abstraction was designed to identify selected patients that may have died as a result of a medical error. An arbitraty target of 50 patients who died unexpectedly was set.

All patients who were admitted into hospital following ED attendance and who died within 72 hours but were not admitted to HDU/ICU were identified by interrogating the participating centers’ electronic database. From the patients identified by database interrogation, 100 from each participating site were randomly selected for initial abstraction. Two chart abstractors (emergency physicians, including one consultant), blinded to each other, reviewed the medical charts to select those patients who may have died unexpectedly. At this stage, patients with limitations of care in place, and patients whose charts were incomplete, were identified and excluded. In cases where the two abstractors disagreed, the opinion of another pair of abstractors was sought. In the absence of a clear ‘do not attempt resuscitation’ order or written decisions regarding limitations of care, the adjudication of the status was made by abstractors upon age; past medical history; comorbidities; quality of life prior to and expected quality of life following illness; and severity of initial presentation. If this status could not be made upon chart review, the cases were excluded. For example, an older patient with dementia living in a nursing home had limitation of care; and a young patient with massive intracranial hemorrhage that could not be treated surgically was excluded because his early death would have been expected.

In the final part of the process of case identification, the medical charts of all included patients were then reviewed by two trained experts. Medical chart information included medical, nursing and handover records; requested, completed and reviewed investigations; and prescribed and administered treatments, for ED and inpatient ward admissions. All ED medical and nonmedical charts are computerized and inpatient charts were not. The preventability of the death was graded by the experts on a 1 to 5 preventability scale (1) very unlikely, 2) unlikely, 3) uncertain, 4) likely, 5) very likely), that was later dichotomized into 1 to 2) unlikely and 4 to 5) likely. In cases of disagreement or uncertainty, consensus was sought between the two experts. When no consensus was reached, the opinion of a third expert, (a professor, chairman) was sought. Following adjudication of the primary endpoint, the two experts reported all suspected medical errors and process breakdowns according to ED process detailed in the secondary endpoint section. Following identification of patients who unexpectedly died, the target of 50 was not met. We therefore selected all charts rather than a random selection, from the largest center (Pitié-Salpêtrière), which provided 155 extra charts for the initial abstraction, making a total of 555.

All charts abstractions were conducted following recommendations made by Kaji *et al*. [15], including training of the abstractors, explicit definition of the inclusion criteria (unexpected death) and endpoint (preventability of death), definition of variables, standardized abstraction forms, regular meetings with the principal investigators and abstractors or experts, blinding abstractors to each other and the testing of inter-rater agreement. Due do the selection process and our inclusion criteria, abstractors could not have been made blind of the final outcome (death).

### Analysis

Qualitative variables are presented as number (percentage), continuous variables as mean (standard deviation (SD)) or median [25th to 75th interquartile range (IQR)] if non-normally distributed. Confidence intervals (CI) were calculated using the exact method. We used Cohen’s kappa scores to assess agreement between the chart abstractors for the inclusion criterion of ‘unexpected death’ and between the experts for the adjudication of the primary endpoint. A kappa >0.6 was considered as a substantial agreement, and almost perfect if >0.8 [16]. All analyses were performed using SPSS software (IBM Corp., Armonk, NY, USA).

## Results

**Figure 1:**
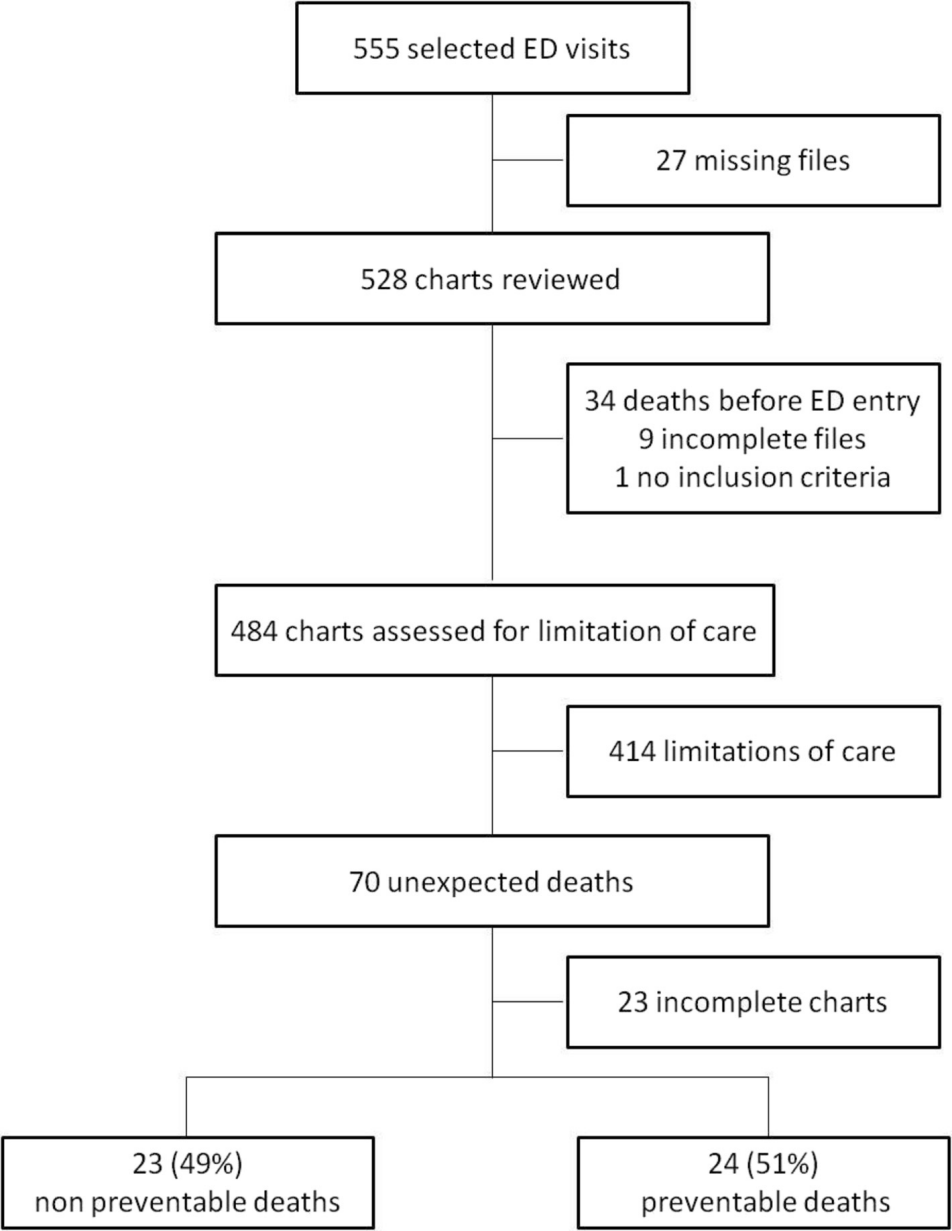
**Flow chart.** ED, emergency department.

During the study period, there were 1,134,032 visits to the four EDs, with 208,549 (18%) patients being admitted from the hospital. Among them, a total of 1,279 admitted patients died within 72 hours without having been admitted to an ICU or HDU. Mean age was 79 years (SD 14), and 51% were men. Demographics and baseline characteristics are reported in Table 1. We retrieved the charts of 555 patients with early death (Figure 1). After exclusion of patients with incomplete charts, missing files and deaths before ED attendance (for example died en route), 484 charts were assessed for limitation of care by the two chart abstractors. Agreement was excellent between the abstractors with a kappa score of 0.81 (95% CI 0.72 to 0.90). Of the 70 patients that died unexpectedly, 23 were noted to have complete ED records but incomplete data on their subsequent hospital stay to adjudicate preventability of death at the second abstraction, and were therefore excluded, leaving 47 for analysis of the primary endpoint. Twenty-four of 47 patients that sustained unexpected death died due to medical error, giving a rate of preventable death of 51%. The agreement between the experts was excellent, with a kappa score of 0.87 (95% CI 0.7 to 1.0). The main characteristics of the 47 charts reviewed by the experts are summarized in Table 1. The most common process breakdowns were incorrect choice of treatment (in 22 (47%) patients), failure to order appropriate diagnostic tests (in 18 (38%) patients), incorrect choice of admission ward (in 12 (47%) patients) and incorrect triage (in 11 (45%) patients).

**Table 1:** **Included patients with early death**

	**All patients**		**Unexpected deaths**	
**Characteristic**				
N	484		47	
**Age (years), mean (SD)**	79	(14)	79	(14)
**Sex male, N (%)**	247	(51%)	32	(68%)
**Center**				
Pitié-Salpêtrière	231	(47%)	25	25
Bichat	90	(19%)	13	(14%)
Ambroise Paré	88	(18%)	4	(5%)
Saint Antoine	75	(15%)	5	(7%)
**Type of arrival**				
own	23	(5%)	7	(15%)
ambulances	376	(78%)	37	(77%)
medical EMS	85	(18%)	3	(6%)
**Vital parameters**				
systolic BP (mmHg), mean (SD)	122	(36)	119	(29)
diastolic BP (mmHg), mean (SD)	69	(22)	70	(22)
heart rate, mean (SD)	93	(27)	92	(26)
Glasgow Coma Scale, median [IQR]	15	[9-15]	15	[15]
temperature (°C), mean (SD)	36.5	(1.4)	36.8	(1.0
**Limitation of care**	70	(14%)	0	(0%)
**Preventability of death**				
very unlikely			10	(21%)
unlikely			13	(27%)
likely			15	(32%)
very likely			9	(32%)

SD, standard deviation; EMS, emergency medical services; BP, blood pressure; IQR, interquartile range.

Death occurred in the ED or ED observational unit in 26 cases (55%), in the medical ward in 19 cases (40%), and in the operating room in four cases (9%).

The details of the main medical errors that may have contributed to the death of the patients are listed in Table 2. Among the 24 patients who had a preventable death, we counted a total of 54 errors - a median of two per patients. A severe delay or the absence of detection and recommended treatment for severe sepsis were the main medical errors in nine patients (38%). Undertriage or under-recognition of acutely ill patients that required close monitoring was considered to be the main error that contributed to the death of the patients in four cases (16%). The experts found that a provider other than the emergency physician was involved in a fatal error in seven cases: namely an orthopedic surgeon (n = 3), a triage nurse (n = 2) and an ICU physician (n = 2).

**Table 2:** **Preventable unexpected death**

**Patient**	**Age**	**Main medical errors**	**Provider**	**ED diagnosis**	**Cause of death**
1	87	Denial of ICU admission	ICU physician	Sigmoid volvulus	Sigmoid volvulus
2	79	wrong dose of opioids analgesic	Orthopedist surgeon	Urinary retention	Opioid intoxication
3	81	No timely treatment of acute coronary syndrome	Emergency physician	Myocardial infarction	Myocardial infarction
4	79	Pacemaker has not been monitored after a syncope	Emergency physician	Syncope	Cardiac arrest
5	83	No fluid resuscitation no antibiotics	Emergency physician	Fatigue	Severe sepsis
6	80	No treatment of congestive heart failure and no blood transfusion	Emergency physician and orthopedic surgeon	Hip fracture	Congestive heart failure
7	87	No control of hyperkaliemia	Emergency physician	Metabolic acidosis	Cardiac arrest
8	83	Undertriage on arrival	Triage nurse	Intracranial hemorrhage	Intracranial hemorrhage
9	43	No chest X-ray before chest drainage	Emergency physician	Respiratory failure	Hemothorax
10	77	No fluid resuscitation and wrong antibiotic administration	Emergency physician	Severe sepsis	Severe sepsis
11	53	No fluid resuscitation and delay in antibiotic administration	Emergency physician	Urinary tract infection	Severe sepsis
12	63	No reheating	Emergency physician	Hypothermia	Hypothermia
13	71	No head CT performed	Emergency physician	Seizure	Intracranial hemorrhage
14	85	No fluid resuscitation no antibiotics	Emergency physician	Severe sepsis	Severe sepsis
15	61	Delay in performing ECG and treatment of acute coronary syndrome	Emergency physician	Acute coronary syndrome	Acute coronary syndrome
16	74	No monitoring and no correction of hypokaliemia	Emergency physician	Ketoacidosis	Hypokaliemia
17	70	No fluid resuscitation	Emergency physician	Pneumoniae	Severe sepsis
18	54	No fluid resuscitation	Emergency physician	Sepsis	Severe sepsis
19	85	No diagnosis and treatment of acute heart failure	Orthopedist surgeon	Hip fracture	Heart failure
20	63	Insufficient fluid resuscitation and denial of ICU admission	Emergency physician and ICU physician	Upper GI bleed	Upper GI bleed
21	87	No fluid resuscitation no antibiotics	Emergency physician	Arthritis	Severe sepsis
22	77	No fluid resuscitation no antibiotics	Emergency physician	COPD exacerbation	Severe sepsis
23	78	No fluid resuscitation and delay in antibiotic administration	Emergency physician	Severe sepsis	Severe sepsis
24	57	Undertriage on arrival and denial of ICU admission	Emergency physician and triage nurse	Heart failure	Heart failure

ED, emergency department; ICU, intensive care unit; CT, computed tomography; ECG, electrocardiography; GI, gastrointestinal; COPD, chronic obstructive pulmonary disease.

## Discussion

In this retrospective study, we found that more than half of early unexpected death after ED visit are related to a medical error and could have been avoided. This is to our knowledge the first study that qualitatively analyzed ED patients with in-hospital early unexpected death after ED attendance. Previous works by Sklar *et al*. and Kefer *et al*. included only discharged patients [9,10]. They retrospectively reviewed the charts of 58 and 33 unexpected deaths, respectively and reported unexpected death rates of 15 to 30 per 100,000 patients. The rate of early unexpected death in our sample was 85 per 100,000 admissions, which is significantly higher than previously reported. This increase may be related to the higher acuity of inpatients compared to outpatients. However, the rate of preventable unexpected deaths in our sample is similar to the one of Sklar *et al*. (50 to 60%), confirming that medical errors are common in cases of unexpected death after ED visits.

We used a predefined list of common steps in the process of care in the ED, ranging from ED entry (that includes prehospital and handover) to admission in the ward or the operation room. Similarly to Kachalia *et al*. [14], we found that a failure to order appropriate diagnostic tests in the ED was one of the most common process breakdown (occurring in 18% of preventable deaths), along with an incorrect choice of treatment (47%).

One particular breakdown emerged repeatedly: the absence of recognition or absence of timely treatment of severe sepsis state, which occurred in nine patients (38%). As shown in Table 2, the insufficient fluid resuscitation or absence of antibiotics therapy may have lead in many cases to a fatal outcome. This theme was not reported in previous studies that retrospectively qualitatively analyzed serious AEs or death [9,10,14]. One possible explanation of this discrepancy is that the studies were performed prior to Rivers’ work on early goal-directed therapy and the inception of the Surviving Sepsis Campaign, which highlighted the need for early recognition and treatment of severe sepsis [17,18]. Since during that period there was no definition of delay for sepsis care, chart abstractors may not have identified care as being delayed. Our study emphasizes the importance of rapid diagnosis of sepsis, and fluids resuscitation. The means to reduce the incidence of this type of errors are varied, and can be human based (for instance enhancing continuous medical education or utilization of simulation-based teaching) or system based (for instance introduction of standard treatment protocols or checklists).

### Limitations

The design of our study has some limitations. The inclusion process was performed electronically based on hospital discharge status. We therefore did not include patients that were discharged from the ED, or before 72 hours. There may have been some patients in that group who died within three days but elsewhere. Data regarding the circumstances of a death outside of our centers but within three days of ED attendance and discharge are clearly not available from the medical record. This group represent a potential source of bias, as errors could have been made that led to their discharge and subsequent death, and these errors cannot be included in our study. Similarly, we did not include those who died after having been transferred to the intensive care unit or to a hospital other than our four centers. A further source of potential bias is that the experts were aware of the fatal outcomes. It has been reported than knowing the outcome can alter the opinion of the reviewer [19]. A relationship between the severity of the outcome and judgments of medical errors has been described. In our study, this bias may have been in favor of a higher rate of medical errors. The very strong agreement between the experts, blinded to each other for the adjudication of the primary endpoint, may be seen as an argument for the validity of our process, although we cannot exclude the fact that both reviewers were biased simultaneously in the same way. The retrospective nature and data collection method of chart reviews represent another limitation and there is an inherent limitation in the subjective nature of adjudication of medical error, as definitions of error or good medical practice are open to regional variation and interpretation. Again, we endeavor to limit this by having a robust adjudication process, which after testing showed high inter-rater reliability.

Finally, the small size of our sample of patients with unexpected death precludes any accurate estimation of the rate of preventability: the 95% CI ranges from 37% to 65%.

## Conclusions

In summary, more than half of early unexpected death after ED visits are related to a medical error and could have been prevented. Underdetection of severe sepsis was a major cause of medical error in our sample.

## Key message

- Prevalence of medical errors among admitted patients who had early unanticipated death after ED visit is unknown.
- In our study, we found that the rate of unanticipated death within 72 hours of ED visit is 85 per 100,000 admissions.
- Half of these deaths were expertised as caused by medical errors
- The most common cause of medical error was a severe delay or the absence of detection and recommended treatment for severe sepsis.

## Abbreviations

AE: adverse event
CI: confidence interval
ED: emergency department
HDU: high dependency unit
ICU: intensive care unit
IQR: interquartile range
SD: standard deviation
